# Oral and maxillofacial manifestations of COVID-19

**DOI:** 10.3205/dgkh000614

**Published:** 2026-01-12

**Authors:** Pooja Palwankar, Jaganbabu Palaniappan, Kavitha Kuttappan, Ruchi Pandey, Shalu Verma, Amit Bhardwaj, Karthik Shunmugavelu

**Affiliations:** 1 Department of Periodontics and Oral Implantology, Santosh Dental College and Hospital, Ghaziabad, Uttar Pradesh, India; 2 Department of Dental surgery, Govt Tiruvannamalai Medical College and Hospital, Tamilnadu, India; 3 Department of Dental surgery, Govt Vellore Medical College and Hospital, Vellore, Tamilnadu, India; 4 Department of Periodontology, Manav Rachna Dental College, SDS, MRIRS, Faridabad, Haryana, India; 5 Department of Pediatric and Preventive Dentistry, Faculty of Dental Sciences, SGT University, Gurugram, Haryana, India; 6 Department of Periodontology and Implantology, Faculty of Dental Sciences, SGT University, Gurugram, Haryana, India; 7 Department of Dentistry, PSP medical college hospital and research institute Tambaram Kanchipuram, Tamilnadu, India

**Keywords:** COVID-19, oral manifestation, maxillofacial manifestation

## Abstract

An overview of oral and maxillofacial manifestations associated with COVID-19 is provided. The symptoms range from white, red and mixed inflamed mucosal areas, necrosis, swelling, ulcers, vesicle, bulla, pustule, pigmentation, depapillated and fissured tongue and bleeding in the ulcers. Pre-COVID symptoms included complete loss of taste, along with reduced sense of taste and alterations in the taste perception.

## Abstract (de)

Es wird ein Überblick über orale und maxillofaziale Manifestationen im Zusammenhang mit COVID-19 gegeben. Die Symptome reichen von weißen, roten und gemischt entzündeten Schleimhautbereichen, Nekrosen, Schwellungen, Geschwüren, Bläschen, Pusteln, Pigmentierung, depapillierter und zerklüfteter Zunge, Erosion und Blutungen in Ulcera. Zu den Präsymptomen gehören der vollständige Verlust des Geschmacks, ein vermindertes Geschmacksempfinden oder Veränderungen der Geschmackswahrnehmung.

## Introduction

Due to the COVID-19, there were also seen some oral and maxillofacial manifestations like white areas, red areas, white and red mixed areas, pigmentation, haemorrhage, necrosis, swelling, vesicle, bulla, pustule, ulcers, pigmentation, fissured tongue, depapillated tongue, inflammation and bleeding in the ulcers and erosion. These disorders are in turn made to verify and detect the patients whether they are infected by COVID-19 [1]. Mostly all the above symptoms were seen in tongue, palate and labial mucosa [2]. The easy and fast spread of COVID-19 is due to SARS-CoV 2. Diagnostic laboratory method included reverse transcription polymerase chain reaction for investigating patients infected with sub-acute COVID-19 [3]. And mostly the confirmed and suspected patients who were infected with COVID-19 cases were having many manifestations like cutaneous, oral and maxillofacial manifestations [4], [5]. 

## Overview about oral and maxillofacial manifestations at COVID-19

The symptoms range from mild inflammation to severe damages like white areas, red areas, white and red mixed areas, inflammation, erosion, pigmentation, swelling, vesicle, bulla, haemorrhage, necrosis, pustule, ulcers, fissured tongue, depapillated tongue, and bleeding in the ulcers [6]. The following distinctions can be made:

- Aphthous ulcer: The ulcers which are formed due to vitamin deficiency and iron in the body and are white in colour are called aphthous ulcers [7].
- Herpetiform: The ulcers that are formed on tongue and are found in bunches are called herpetiform ulcers [8].
- White lesions: When the thickness and volume increases of keratin layer due to friction or any immunological disorders on the tongue, it forms ulcers which are called white lesions as they are white in colour [9].
- Red lesions: Red lesions are on both the tongue and the palate. The red lesions are also called erythroplasia as they are in red colour and has a velvety texture. These are not found in clusters and are also found on the mouth floor [10].
- Erythema-multiforme like lesions: Due to the excess use of antigens, the mucosa and the skin may cause lesions called Erythema multiforme (EM) [11], [12], [13], [14].
- Angina bullosa like lesions: Angina bullosa haemorrhagica (ABH) is a lesion which increase in size and burst and release the blood in the mucosa and epithelial layer and causes pain in the oral cavity [15].
- Melkersson-Rosenthal syndrome: The rare disorder that shows its effects on neurons of the upper lip and also develops a fissured tongue with the folds in it and also it causes face swelling and facial paralysis is Melkersson-Rosenthal syndrome [16].
- Atypical Sweet syndrome: The syndrome in which the lesions occur in both oral cavity and skin that causes more inflammation and also which is associated with pyrexia is Atypical Sweet syndrome [17], [18].
- Kawasaki like disease: In Kawasaki like disease, main pathognomonic feature includes strawberry tongue with bumpy look and fungiform papillae followed by dryness, peeling, fissuring, haemorrhagic areas, vertical cracking and diffuse changes [19].
- Necrotizing periodontal disease: Oral condition of necrotic category includes necrotizing gingivitis, acute necrotizing ulcerative periodontitis, necrotizing stomatitis and cancrum oris. In these conditions, common features are necrosis of gingiva, ulcerations seen in the interdental region, followed by osteonecrosis [20].
- Vesicles: A vesicle is a small bullae which may or may not be equal to 1 cm in diameter characterised by elevation and fluid-filled lesion which in turn is covered by epithelium. This fluid accumulation can occur either outside the epithelium called intraepithelial vesicle or below the epithelium called subepithelial vesicle [21].
- Pustules: Common sites of involvement are buccal mucosa, hard palate, vestibule, gingiva, soft palate and lateral borders of tongue in case of multiple pustule occurance [22].
- Mucositis: Most common etiology for mucositis are anthracyclines, alkalytaing agents, mTOR inhibitors, and antimetabolites which might lead to erythematous areas in conjunction with edema and ulcerations [23], [24], [25].
- Petichiae: Hemorrhages which might occur pin-point appearances in sub-cutaneous or sub-mucosal are called petechiae [26].
- Post inflammatory pigmentation: Auto-immune diseases such as oral lichen planus, oral lichenoid lesions, pemphigoid, pemphigus, graft versus host disease, Steven-Johnson syndrome, and sometimes also periodontal disease might lead to oral post-inflammatory pigmentation resulting in change in colour of the oral mucosa [27], [28], [29].

Descending order of site of involvement includes tongue which accounts for 38%, followed by 26% of labial mucosa and finally 22% of palate. Above mentioned sites present lesions of Kawasaki-like disease, Erythema-multiforme like lesions, drug eruption, oral mucositis, atypical Sweet syndrome, angular cheilitis, Melkersson-Rosenthal syndrome, angina bullosa-like lesions, necrotizing periodontal disease, aphthous stomatitis, candidiasis, vasculitis and herpetiform lesions. Symptomatic cases accounted for 68% in relation to oral and maxillofacial lesions. The lesions were more noted in males of 51% followed by 49% of females. Severe cases of COVID-19 with oral manifestations were observed in geriatric population. Risk factors included poor oral hygiene, medically compromised conditions, emotional stress, immune compromised conditions, vasculitis, and hyper-inflammatory response [2]. The main common symptom seen in most of the patients was related to the taste disorder [30]. Other common symptoms which are seen in COVID-19 patients were alike other viral infections like myalgia, arthralgia, sore throat, cough, fever, headache, excess sputum production and dyspnoea. Oral and maxillofaciallary manifestations might lead to functional disorders resulting in gastrointestinal symptoms such as nausea, vomiting, tremors, anorexia and diarrhoea [31], followed by dermatological manifestations and neurological dysfunctions [32]. Pre-COVID symptoms included complete loss of taste, along with reduced sense of taste and alterations in the taste perception. Altered sense of taste is mentioned as Dysgeusia, diminished taste sensation is mentioned as Hypogeusia and complete absence of taste is defined as Ageusia [33], [34], [35], [36], [37].

## Conclusion

Due to the lack of immunity, lack of personal hygienic measures and also the less confidence in recovery, the COVID-19 in effected patients was proved to be fatal [38]. As there was COVID-19 in many patients, the symptoms seen were bleeding in the ulcers, angular cheilitis and pressure ulcers and this ultimately raised the disease progression which also caused death to many patients [39].The most commonly observed intra-oral conditions are dry mouth, appearance of vesiculobullous lesions and alterations in taste perceptions. As there was increase in cases of COVID-19 patients, the oral and maxillofacial manifestations were seen in mostly all the patients and thus, dentists were needed to play their roles in the early diagnosis of the infections caused by the coronavirus [40]. Along with the SARS-CoV-2, it’s new mutants were also found in many patients like Omicron which were changing its infrastructure and were given a shape to the highly contagious virus. So for these kinds of mutants, we need to conduct studies more and more and find the virus and its effects on the oral cavity. The patients who are infected with COVID-19 and the older patients should be first prioritized by the examiners or dental professionals so that there will be timely treatment done so that it increases the quality of the patient’s life [41]. The patients who have serious medical conditions such as diabetes, chronic lung diseases or any heart conditions and also the patients with poor oral health are at high risk for getting infected due to COVID [42]. Although the recovery was seen in the patients with the poor oral conditions, but it took some time for them to recover [43]. The above said oral and maxillofacial manifestations like ulcers and lesions were caused due to COVID and to recover, firstly proper hygienic conditions like oral hygiene have to be followed to improve the health of the patient effected by COVID-19 [44].

## Notes

### Authors’ ORCIDs

- Shunmugavelu K: https://orcid.org/0000-0001-7562-8802
- Bhardwaj A: https://orcid.org/0000-0003-2194-6455
- Kuttappan K: https://orcid.org/0009-0003-1410-3684
- Verma S: https://orcid.org/0009-0008-4209-7564
- Palanaiappan JB: https://orcid.org/0009-0005-1767-7741
- Palwankar P: https://orcid.org/0000-0001-7537-585X
- Pandey R: https://orcid.org/0000-0001-9667-0778

### Funding

None. 

### Competing interests

The authors declare that they have no competing interests.
